# Etymologia: *Sarcocystis nesbitti*

**DOI:** 10.3201/eid1912.ET-1912

**Published:** 2013-12

**Keywords:** etymologia, Sarcocystis nesbitti, protozoa, Friedrich Miescher, snakes

***Sarcocystis nesbitti* [sahrʺko-sisʹtis nez-bitʹē]**

In 1843, Swiss scientist Friedrich Miescher found “milky white threads” in the muscles of a mouse, which for years were known as“Miescher’s tubules.” In 1882, Lankester named the parasite *Sarcocystis*, from the Greek *sarx* (flesh) and *kystis* (bladder). Scientists were unsure whether to classify the species as protozoa or as fungi because only the sarcocyst stage had been identified. In 1967, crescent-shaped structures typically found in protozoa were seen in sarcocyst cultures, and it was determined to be a protozoan, a close relative of *Toxoplasma* spp. In 1969, A. M. Mandour described a new species of *Sarcocystis* in rhesus macaques, which he named *Sarcocystis nesbitti*, after Mr. P. Nesbitt, who saw the trophozoites in stained smears. Snakes are now known to be the definitive hosts of *S. nesbitti*, and several primates, including humans, can be intermediate hosts.
